# “Era pro neném”: o direito à maternidade e considerações sobre gestantes em situação prisional no Brasil

**DOI:** 10.1590/0102-311XPT054525

**Published:** 2025-07-04

**Authors:** Marina Maria Ribeiro Gomes da Silva, Tatiana Wargas de Faria Baptista

**Affiliations:** 1 Instituto Nacional de Saúde da Mulher, da Criança e do Adolescente Fernandes Figueira, Fundação Oswaldo Cruz, Rio de Janeiro, Brasil.

A quem cabe o direito à maternidade no Brasil? A quais mães os marcos legais que tratam dessa pauta se destinam? Essas questões permeiam o livro de Luciana Simas Chaves de Moraes ^1^, decorrente de sua tese, defendida em 2018, e que discutiu o direito de mães em situação prisional, analisando o modelo jurídico do país para proteção de gestantes e lactantes privadas de liberdade.

Trata-se de um estudo robusto que aproxima diferentes campos e debate a interface “saúde, direito e ética”, diante do superencarceramento e das violações de direitos do Estado com a população carcerária brasileira. Com sua trajetória como advogada e feminista, a pesquisadora se aprofunda no levantamento de leis e políticas, resultantes de reivindicações que evidenciam a urgência do enfrentamento à vulnerabilização de mães encarceradas.

Na defesa pelo desencarceramento, mais indagações são identificadas: até quando mulheres grávidas e mães seguirão com crianças no cárcere? Como assegurar direito à saúde a elas? A invisibilização dessa agenda é tensionada, reforçada pela crítica à lógica patriarcal e culpabilizante nas medidas desencarceradoras e pela escuta a gestantes privadas de liberdade, enfatizando a importância do tema para a saúde pública.

Recentemente, aumentaram os estudos sobre cárcere, oriundos de diferentes áreas. E alguns, numa perspectiva abolicionista, problematizam a estruturação das prisões para receber mães e crianças, defendendo o desencarceramento delas. O livro fortalece essa defesa e constrói caminhos metodológicos transdisciplinares para discutir maternidade na Modernidade, direitos humanos, criminologia, equidade de gênero e saúde nas prisões, expondo fragilidades de um sistema prisional que segrega e exclui.

As referências sobre existir no cárcere apresentadas revelam que mães e filhos não têm o direito humano à saúde nas prisões brasileiras, com violações, falta de informação, subfinanciamento e subnotificações, de forma que o Estado sustenta uma instituição que inviabiliza a vida. Adiciona-se ainda um contexto marcado por estigma, discriminação e preconceito com a população carcerária, sendo a prisão, para a autora, uma tecnologia de repressão criminal e sanitária, em que a pena é aplicada como cura, numa lógica punitiva para reparar pessoas.

Mas a prisão é espaço de reparação? Moraes avalia haver no cárcere a perpetuação do estado de polícia e não de direito, que não reconhece pessoas na sua diversidade como sujeitos, violando a dignidade, liberdades fundamentais e direitos humanos. E estamos falando de 748.009 pessoas presas nessas condições no Brasil em 2017, segundo o Departamento Penitenciário Nacional (Depen), tornando-se a terceira maior população carcerária mundial. A taxa de ocupação dos presídios então era de 197,4%, com o dobro de pessoas diante das vagas. Como problematizado no livro, apesar da superlotação amplificar violações, não cabe reivindicar vagas, mas encarar o superencarceramento no país “*como um problema estrutural mais amplo, vinculado a um processo de criminalização da miséria*” (p. 26).

O aprisionamento de mulheres é ainda maior, aumentando em 800% de 2000 para 2016 no Brasil, segundo o Ministério da Justiça. A maioria delas cumpre pena em regime fechado, acusada de crimes sem violência, geralmente tráfico de drogas, e tem idade reprodutiva, sendo 50% jovens e 62% mulheres pretas e pardas. A sobrerrepresentação de mulheres negras nas prisões reproduz séculos de seletividade penal, associada historicamente ao racismo estrutural. Os impactos para as mais pobres envolvem as famílias, já que, em muitos casos, elas que sustentam financeira e emocionalmente o lar, gerando dificuldades materiais e afetivas.

Moraes explica que a prisão não resguarda especificidades de gênero, com inadequação de banheiros para mulheres e falta de roupas íntimas, absorventes e assistência em saúde. O machismo está no contexto jurídico penal, sendo gênero, numa abordagem interseccional, um dos indicadores para opressões e desigualdades.

Dados do Infopen Mulheres/Depen ^2^ mostram que, em 2018, eram 536 gestantes e 350 lactantes presas no país, das quais 50% estavam em “celas adequadas” para o Ministério da Justiça. Ou seja, o Estado confessa que mulheres grávidas e lactantes estão com crianças em espaços inadequados. E a autora questiona: existe espaço adequado para gestação e maternidade nas prisões?

A publicação situa a maternidade como um direito social previsto na *Constituição Federal* de 1988 e reitera a responsabilização estatal para garantir a dignidade da vida de grávidas e lactantes encarceradas. Assim, assegurar a quem opte por maternar que o faça de forma benéfica para si e para a criança nas prisões é reconhecer a coexistência de diferentes sujeitos de direitos e que o Estado deve possibilitar maternidade segura em qualquer espaço.

Um risco eminente ao direito à maternidade no cárcere, numa sociedade misógina e patriarcal, é a priorização das crianças em detrimento das mulheres, como se a presa grávida ou mãe com criança precisasse se adequar ao modelo de “mãe ideal”, vigiada pelo sistema prisional e outras presas. Para Moraes: “*A gestação e o nascimento dos filhos na prisão podem representar uma nova privação*” (p. 41). Nessa linha, Braga ^3^ denomina de “hipermaternidade” esse excesso de disciplinamento da maternidade encarcerada e identifica que a impossibilidade dessas mulheres frequentarem atividades de remissão da pena para cuidar exclusivamente dos filhos aumenta sua vulnerabilidade. Há, segundo a autora, um cunho moral no debate sobre o direito à maternidade nas prisões, associado a uma questão de gênero mais ampla da sociedade: a atribuição do cuidado com as crianças às mulheres.

O estudo analisou ainda decisões e normas vigentes em âmbito federal relativas ao direito à maternidade nas prisões. Entre os marcos federais pesquisados sobre o direito à saúde para mulheres e filhos no cárcere estão: a Política Nacional de Atenção Integral à Saúde das Pessoas Privadas de Liberdade no Sistema Prisional; a Política Nacional de Atenção às Mulheres em Situação de Privação de Liberdade e Egressas do Sistema Prisional; e a Política Nacional Integrada para a Primeira Infância. Internacionalmente, foram consideradas as Regras de Bangkok, da Organização das Nações Unidas, que regula medidas não privativas de liberdade para mulheres.

A realidade das prisões explicita, no entanto, um descompasso com as melhorias estabelecidas nesses documentos. A precariedade do atendimento no pré-natal e parto para gestantes privadas de liberdade é notada em relatos de uso de algemas, desumanização, além de entraves para medidas desencarceradoras. “*A prisão expõe um apartheid social, em limites que estão muito além dos seus muros. Buscar alternativas para que a sociedade possa reverter esse quadro de exclusão e abandono implica em se alterar valores morais e sociais, com base no respeito universal*” (p. 141).

A partir da análise qualitativa das decisões sobre gestantes em audiências de custódia de 2015 a 2017, disponibilizadas pelo Tribunal de Justiça do Estado do Rio de Janeiro, a autora selecionou mulheres que respondiam processos criminais em liberdade provisória ou prisão domiciliar. O delito da maioria foi furto, apontando baixa periculosidade. Em um dos casos, a entrevistada descreve a violência ao ser presa, mesmo o policial sabendo de sua gravidez: “*Tudo bem, eu errei, mas, poxa, tem uma criança na minha barriga, sabe? O policial foi agressivo comigo* (…) *Eu furtei coisa de criança, que era pro neném: xampu, sabonete* (…)” (p. 195).

As narrativas das entrevistadas evidenciam desrespeito, sofrimento, falta de informação e negligências do Estado. Mostram vulnerabilizações oriundas de restrições impostas com medidas não privativas, como acontece na prisão domiciliar ao obrigar que a ré permaneça em casa, sem poder sair para trabalhar, e violências no atendimento no pré-natal, representando violações de direitos com repercussão nas crianças.

O livro é uma importante contribuição para estudos com/sobre/para pessoas privadas de liberdade, apresentando aspectos éticos e desafios enfrentados, como dificuldades no contato para entrevistas, desconfiança das entrevistadas e medo de serem discriminadas. Definitivamente, reforça que prisão não é lugar para gestantes e mães com crianças, nos implicando nessa reflexão, e que medidas alternativas ao encarceramento necessitam de aperfeiçoamento permanente. Os avanços nas normativas pelo direito à maternidade nesse contexto resultam de denúncias de movimentos feministas e de direitos humanos, mas ainda são insuficientes para garantir equidade, sendo urgente romper com discursos autoritários e de repressão e investir na formação dos agentes públicos para uma atuação inclusiva, que reconheça gestantes e lactantes em situação prisional como sujeitos de direito, não apenas nutrizes das crianças.
